# Lung branching morphogenesis is accompanied by temporal metabolic changes towards a glycolytic preference

**DOI:** 10.1186/s13578-021-00654-w

**Published:** 2021-07-17

**Authors:** Hugo Fernandes-Silva, Marco G. Alves, Henrique Araújo-Silva, Ana M. Silva, Jorge Correia-Pinto, Pedro F. Oliveira, Rute S. Moura

**Affiliations:** 1 Life and Health Sciences Research Institute (ICVS), School of Medicine, University of Minho, 4710-057 Braga, Portugal; 2 ICVS/3B’s - PT Government Associate Laboratory, 4710-057 Braga/Guimarães, Portugal; 3 PhDOC PhD Program, ICVS/3B’s, School of Medicine, University of Minho, 4710-057 Braga, Portugal; 4 Unit for Multidisciplinary Research in Biomedicine (UMIB), Institute of Biomedical Sciences Abel Salazar (ICBAS), University of Porto, 4050-313 Porto, Portugal; 5 Department of Microscopy, Institute of Biomedical Sciences Abel Salazar (ICBAS), University of Porto, 4050-313 Porto, Portugal; 6 Department of Pediatric Surgery, Hospital of Braga, 4710-243 Braga, Portugal; 7 QOPNA &, LAQV, Department of Chemistry, University of Aveiro, 3810-193 Aveiro, Portugal

**Keywords:** Metabolism, Respiratory system, Warburg effect, Lactate dehydrogenase, Chicken embryo

## Abstract

**Background:**

Lung branching morphogenesis is characterized by epithelial-mesenchymal interactions that ultimately define the airway conducting system. Throughout this process, energy and structural macromolecules are necessary to sustain the high proliferative rates. The extensive knowledge of the molecular mechanisms underlying pulmonary development contrasts with the lack of data regarding the embryonic lung metabolic requirements. Here, we studied the metabolic profile associated with the early stages of chicken pulmonary branching.

**Methods:**

In this study, we used an ex vivo lung explant culture system and analyzed the consumption/production of extracellular metabolic intermediates associated with glucose catabolism (alanine, lactate, and acetate) by ^1^H-NMR spectroscopy in the culture medium. Then, we characterized the transcript levels of metabolite membrane transporters (*glut1*, *glut3*, *glut8*, *mct1*, *mct3*, *mct4*, and *mct8*) and glycolytic enzymes (*hk1*, *hk2*, *pfk1*, *ldha*, *ldhb*, *pdha*, and *pdhb*) by qPCR. *ldha* and *ldhb* mRNA spatial localization was determined by in situ hybridization. Proliferation was analyzed by directly assessing DNA synthesis using an EdU-based assay. Additionally, we performed western blot to analyze LDHA and LDHT protein levels. Finally, we used a Clark-Type Electrode to assess the lung explant's respiratory capacity.

**Results:**

Glucose consumption decreases, whereas alanine, lactate, and acetate production progressively increase as branching morphogenesis proceeds. mRNA analysis revealed variations in the expression levels of key enzymes and transporters from the glycolytic pathway. *ldha* and *ldhb* displayed a compartment-specific expression pattern that resembles proximal–distal markers. In addition, high proliferation levels were detected at active branching sites. LDH protein expression levels suggest that LDHB may account for the progressive rise in lactate. Concurrently, there is a stable oxygen consumption rate throughout branching morphogenesis.

**Conclusions:**

This report describes the temporal metabolic changes that accompany the early stages of chicken lung branching morphogenesis. Overall, the embryonic chicken lung seems to shift to a glycolytic lactate-based metabolism as pulmonary branching occurs. Moreover, this metabolic rewiring might play a crucial role during lung development.

**Supplementary Information:**

The online version contains supplementary material available at 10.1186/s13578-021-00654-w.

## Background

Pulmonary branching morphogenesis is an intricate process governed by epithelial-mesenchymal interactions and is dependent on complex signaling events. This process occurs throughout the early stages of embryonic lung development and defines the respiratory airway structure [1]. In the chicken, *Gallus gallus*, the primordial lung appears around day 3 of embryogenesis as a protuberance from the primitive foregut [2]. During this process, the mesobronchus grows distally, and the new secondary bronchi sprout laterally into the surrounding mesenchymal compartment [1, 3]. This lateral or monopodial branching is exceptionally similar to the domain branching subroutine characteristic of the mammalian lung system [4, 5]. Moreover, the molecular events underlying the development of the avian respiratory system are highly conserved with the mammalian and point to similar functions [6]. For instance, FGF (Fibroblast Growth Factor), WNT (Wingless-related Integration Site), SHH (Sonic Hedgehog), and Retinoic Acid signaling pathways were described as playing critical roles in chicken pulmonary branching morphogenesis [7–11].

The signaling mechanisms involved in early lung development are quite well studied in several animal models [10, 12, 13]; however, little is known concerning the embryonic lung metabolic needs [14, 15]. As for the mammalian adult lung metabolic requirements, it has been shown that they are achieved through the uptake and catabolism of glucose, which represents the primary fuel to the adult lung tissue [16–18]. The coordination between signaling and metabolism is now emerging as a key concept for understanding developmental processes. Therefore, it is of major importance to investigate how metabolism contributes and is dynamically regulated during animal development [19, 20].

Recent studies have demonstrated that glycolysis can serve additional roles beyond the classical bioenergetics purpose and contribute to shaping embryonic development both in time and space. For instance, during the murine chorioallantoic branching stage, the developing embryo redirects glucose carbon flow into the pentose phosphate pathway by suppressing phosphofructokinase 1 (PFK1) and aldolase; concomitantly, there is an increase in the glycolytic fraction to serve lactate biosynthesis. This study suggests a rewiring of glycolytic metabolism in the whole embryo, and over time, to promote accurate chorioallantoic branching [21]. Moreover, Slaninova et al. showed that loss of NOTCH signaling in Drosophila and human microvascular cells resulted in the downregulation of pivotal glycolytic genes; in opposition, a short pulse of NOTCH signaling stimulates glycolysis and increases lactate production, in detriment of Tricarboxylic Acid Cycle (TCA) activity [22]. Overall, these results revealed that NOTCH signaling promotes a glycolytic shift that resembles the Warburg effect [22]. Furthermore, Drosophila larval neuroblast cells, while proliferating, depend on aerobic glycolysis. However, during cell cycle exit and terminal differentiation, cell metabolism switches from aerobic glycolysis to an OXPHOS-based metabolism [23]. More recently, Bulusu et al. described a glycolytic activity gradient that contributes to Presomitic Mesoderm (PSM) differentiation and development in the mouse embryo [24]. Additionally, it has been shown that there is an FGF/Wnt coordinated glycolytic gradient that regulates cell motility and controls specification, thus contributing to PSM development in the chicken embryo [25].

This study aimed to investigate the metabolic profile underpinning the early stages of pulmonary branching morphogenesis. Chicken embryonic lungs were used to perform ex vivo lung explant culture. Extracellular metabolites associated with glucose catabolism (alanine, lactate, and acetate) were evaluated by ^1^H Nuclear Magnetic Resonance (^1^H-NMR) spectroscopy in the culture medium. The expression patterns/levels of key enzymes and transporters from the glycolytic pathway were assessed by in situ hybridization and qPCR, namely: glucose transporters (*glut1*, *glut3*, and *glut8*), monocarboxylate transporters (*mct1*, *mct3*, *mct4*, and *mct8*), and metabolic-related enzymes such as hexokinase (*hk1* and *hk2*), phosphofructokinase (*pfk1*), lactate dehydrogenase (*ldha* and *ldhb*) and pyruvate dehydrogenase (*pdha* and *pdhb*). Proliferation status was determined by directly assessing DNA synthesis using an EdU-based assay. Additionally, the protein expression levels of LDH were determined by Western blot. Lastly, the respiratory capacity of explants was evaluated by measuring the basal oxygen consumption rate (OCR).

This report describes, for the first time, the temporal metabolic changes that accompany the early stages of chicken lung branching morphogenesis. In our experimental setting, the embryonic chicken lung shows a glycolytic preference with a shift to lactate production as pulmonary branching proceeds.

## Results

### Lung branching morphogenesis is accompanied by temporal metabolite changes

**Fig. 1:**
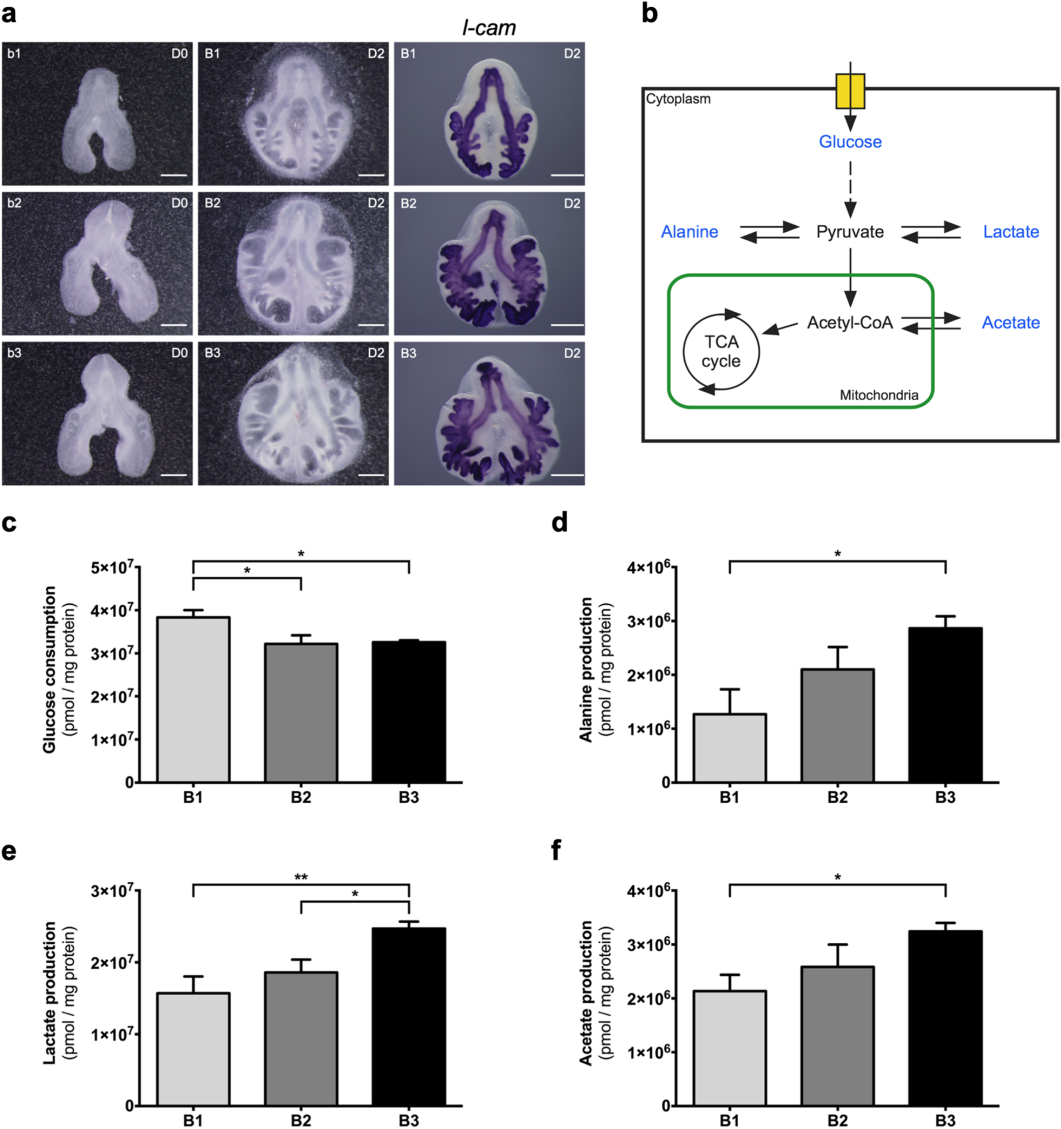
Extracellular metabolite fluctuations throughout early stages of lung branching. **a** Representative examples of embryonic chicken lungs at DO (0 h) (b1, b2, b3) and D2 (48 h) (B1, B2, B3) of explant culture, and corresponding in situ hybridization for *l-cam*, (n = 5/stage). *l-cam* is an epithelial marker, showing active branching formation. Scale bar: 500 µm. **b** Schematic representation of the glucose catabolism pathway and the potential pyruvate destinations. Blue labeling indicates the metabolites that were detected and quantified in the ^1^H-NMR spectroscopy analysis. **c** Glucose consumption, **d** Alanine production, **e** Lactate production, and **f** Acetate production, during 48 h of explant culture. The medium was refreshed at D1 (24 h). Medium samples were collected at D1 (24 h) and D2 (48 h). D0 media samples were used as control. Metabolite consumption or production was calculated following the mathematical formula | (D1-D0) + (D2-D0) |. Metabolite data was normalized to the total amount of protein. Results are expressed as | mean |± SEM (n ≥ 5/stage). One-Way ANOVA and Fisher’s LSD test were performed. Significantly different results are indicated as: *p < 0.05; **p < 0.01

To describe the metabolic alterations that occur during the early stages of chicken pulmonary branching, in vitro lung explant culture was performed using stages b1, b2, and b3 (1, 2, or 3 secondary buds formed per bronchus, respectively) that correspond to the first three branching stages (Fig. 1a); new branches are clearly seen with the epithelial marker *l-cam* (Fig. 1a). Explant culture allows assessing extracellular metabolite fluctuations while in a controlled environment. Briefly, the culture system was performed for 48 h and refreshed at D1 (24 h); medium was collected at D0 (0 h; to be used as reference/control), D1, and D2 (48 h), and then analyzed by ^1^H-NMR spectroscopy. Metabolite production/consumption was calculated following the mathematical formula | (D1-D0) + (D2-D0) |, expressed in pmol, and normalized to the total amount of protein. From the ^1^H-NMR spectra analysis, it was possible to detect the following metabolites: glucose, alanine, lactate, and acetate (Fig. 1b). Additionally, morphometric analysis was performed to assess branching, revealing a gradual increment of the epithelial compartment when comparing between stages (B1 *vs* B2 *vs* B3), which implies an increase in lung branching morphogenesis (Additional file 1: Figure S1).

The ^1^H-NMR results revealed that glucose consumption, by the embryonic lung, decreases from B1 (3.8 × 10^7^ ± 1.7 × 10^6^ pmol/mg protein) to B2 (3.2 × 10^7^ ± 2.0 × 10^6^ pmol/mg protein) (*p* < 0.05) and from B1 to B3 (3.3 × 10^7^ ± 4.7 × 10^5^ pmol/mg protein) (p < 0.05) (Fig. 1c). Alanine is produced (Fig. 1d), revealing a statistically significant increase from B1 (1.3 × 10^6^ ± 4.6 × 10^5^ pmol/mg protein) to B3 (2.9 × 10^6^ ± 2.2 × 10^5^ pmol/mg protein) (*p* < 0.05). Likewise, a progressive upsurge in lactate production is detected (Fig. 1e). Indeed, the production of lactate during branching increases from stage B1 (1.6 × 10^7^ ± 2.3 × 10^6^ pmol/mg protein) and from stage B2 (1.9 × 10^7^ ± 1.8 × 10^6^ pmol/mg protein) to B3 (2.5 × 10^7^ ± 9.7 × 10^5^ pmol/mg protein) (*p* < 0.01 and *p* < 0.05, respectively). Similarly, acetate production (Fig. 1f) increases through lung branching stages, namely from B1 (2.1 × 10^6^ ± 3.0 × 10^5^ pmol/mg protein) to B3 (3.2 × 10^6^ ± 1.6 × 10^5^ pmol/mg protein) (*p* < 0.05).

### Key glycolytic enzymes and transporters are present in the embryonic chicken lung

**Fig. 2:**
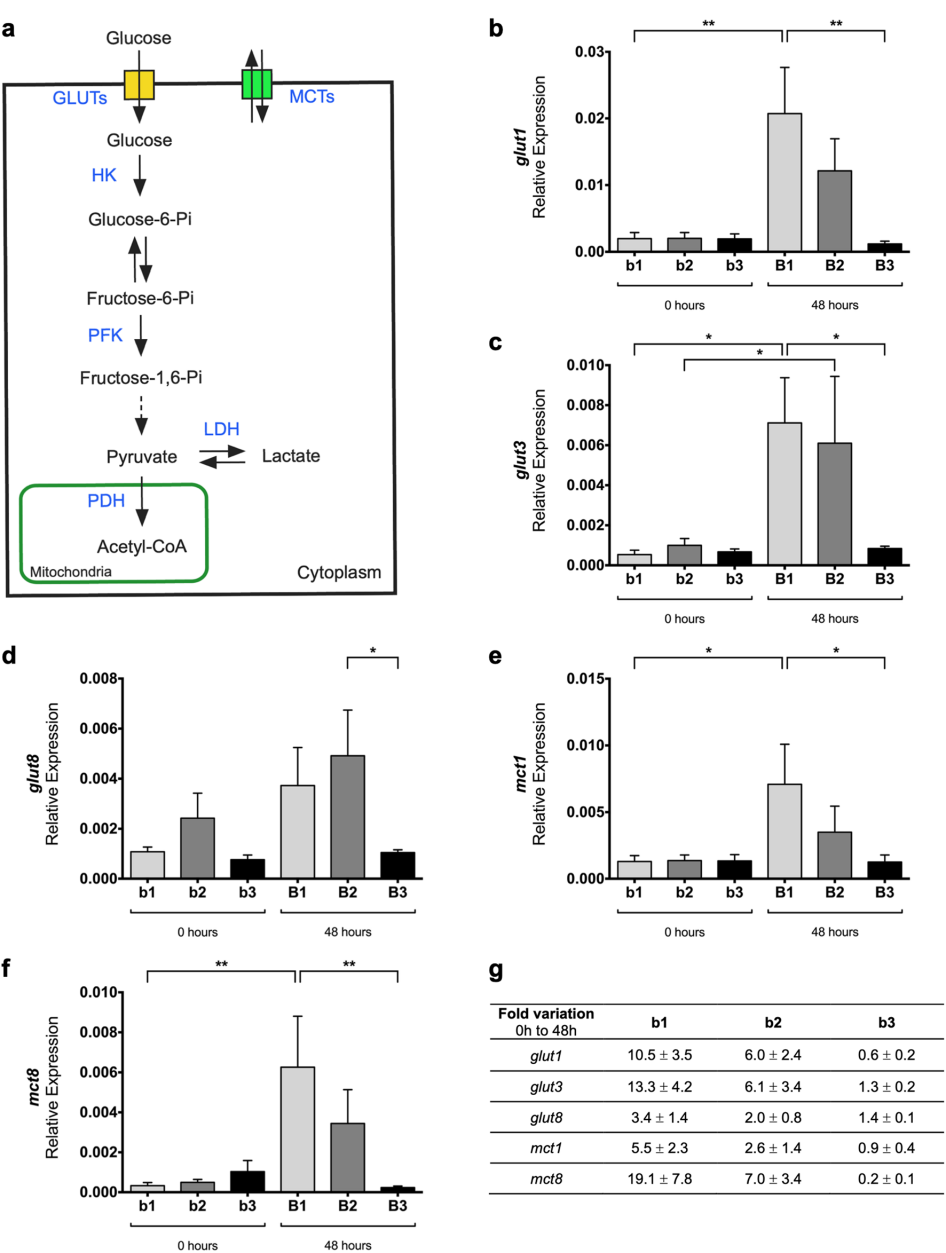
mRNA expression levels of glucose catabolism transporters in early stages of lung branching. **a** Schematic representation of the glucose catabolism pathway. Blue labeling indicates the enzymes and transporters that were evaluated by qPCR. Relative expression levels of **b** *glut1*, **c** *glut3*, **d** *glut8*, **e** *mct1*, and **f** *mct8* in embryonic lungs at D0 (0 h) (b1, b2, b3) and D2 (48 h) (B1, B2, B3) of explant culture. **g** Expression fold variation, from 0 to 48 h of explant culture, for *glut1*, *glut3*, *glut8*, *mct1*, and *mct8*. mRNA expression levels were normalized for both *18 s* and *actin-β* housekeeping genes. Results are expressed in arbitrary units, as mean ± SEM (n ≥ 5/stage/condition). One-Way ANOVA and Fisher’s LSD test were performed. Significantly different results are indicated as: *p < 0.05; **p < 0.01

To characterize the molecular machinery underlying the metabolic variations associated with early branching stages, key enzymes and transporters involved in glucose catabolism were evaluated by qPCR (Fig. 2a). For this purpose, b1 to b3 lung explants were collected at 0 h and after 48 h of culture (Fig. 1a) and assessed for the expression of *glut1*, *glut3*, *glut8*, *mct1*, *mct3*, *mct4*, *mct8*, *hk1*, *hk2*, *pfk1*, *ldha*, *ldhb*, *pdha*, and *pdhb*.

*glut1*, *glut3*, and *glut8* transcripts (Fig. 2b–d) are present in all three pulmonary stages. *glut1* displays an increase in the expression levels from b1 (0 h) to B1 (48 h) with *p* < 0.01 (Fig. 2b). Similarly, *glut3* expression levels (Fig. 2c) increase between b1 and B1 (p < 0.05), and also between b2 and B2 (*p* < 0.05). After 48 h, both *glut1* and *glut3* expression levels decrease from B1 to B3 (*p* < 0.01; *p* < 0.05). Likewise, *glut8* transcript (Fig. 2d) decreases from B2 to B3 (*p* < 0.05).

*mct1* and *mct8* transcripts (Fig. 2e, f) display a similar tendency in the embryonic chicken lung. Both *mct1* and *mct8* exhibit an increase from b1 to B1 (*p* < 0.05; p < 0.01) and then a decrease from B1 to B3 (*p* < 0.05; *p* < 0.01). After 48 h, MCT transcripts behave comparably to GLUTs. *mct3* and *mct4* expression levels were virtually undetectable in the embryonic chicken lung (data not shown).

**Fig. 3:**
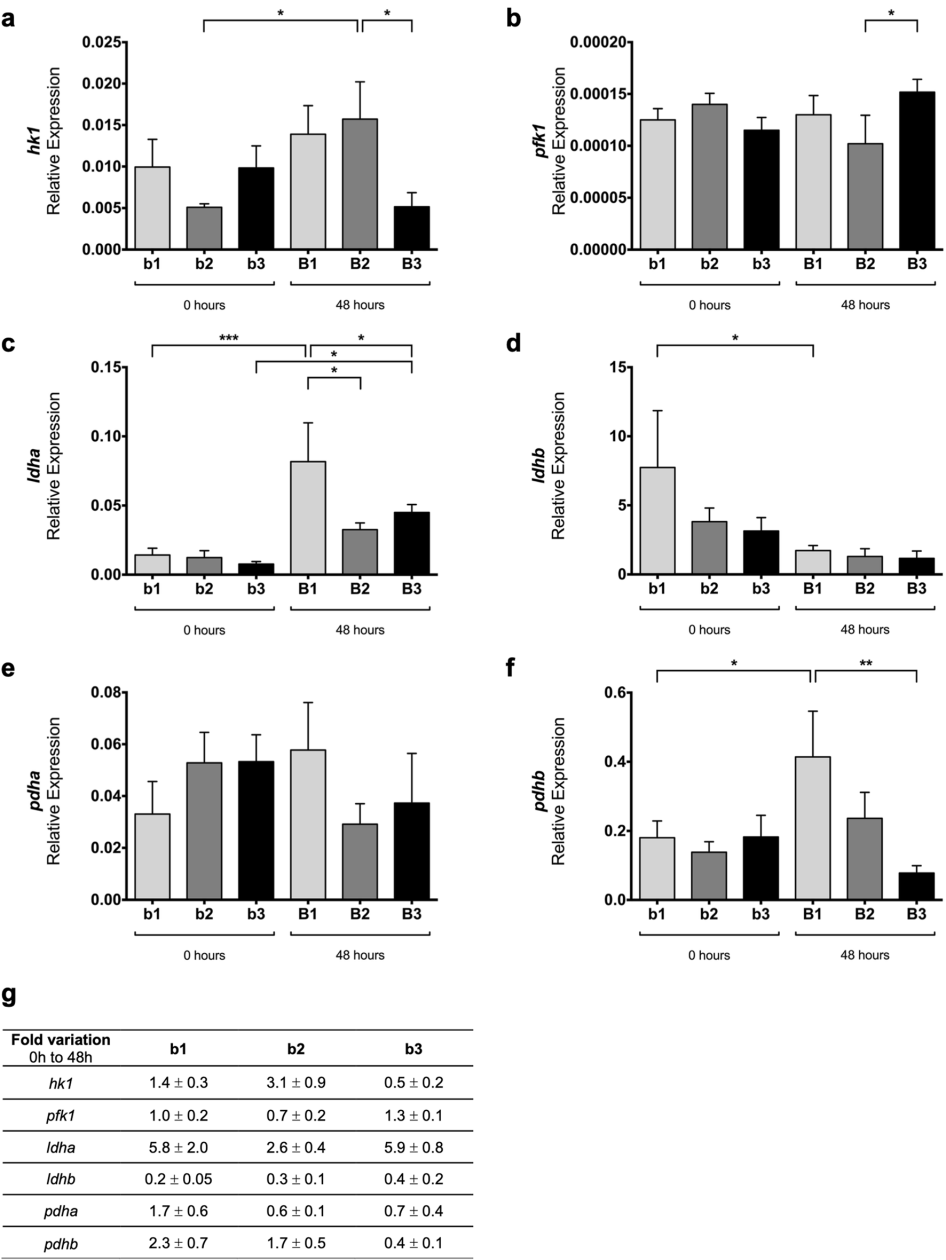
mRNA expression levels of glucose catabolism enzymes in early stages of lung branching. Relative expression levels of **a** *hk1*, **b** *pfk1*, **c** *ldha*, **d** *ldhb*, **e** *pdha*, and **f** *pdhb*, in embryonic lungs at D0 (0 h) (b1, b2, b3) and D2 (48 h) (B1, B2, B3) of explant culture. **g** Expression fold variation, from 0 to 48 h of explant culture, for *hk1*, *pfk1*, *ldha*, *ldhb*, *pdha*, and *pdhb*. mRNA expression levels were normalized for both *18 s* and *actin-β* housekeeping genes. Results are expressed in arbitrary units, as mean ± SEM (n ≥ 5/stage/condition). One-Way ANOVA and Fisher’s LSD test were performed. Significantly different results are indicated as: *p < 0.05; **p < 0.01; ***p < 0.001

*hk1* transcript (Fig. 3a) is present in the three pulmonary stages studied and displays statistically significant differences between b2 and B2 (*p* < 0.05). Also, *hk1* expression decreases from B2 to B3 (*p* < 0.05). *hk2* mRNA levels are practically null in the embryonic chick lung (data not shown).

*pfk1* expression levels are maintained between groups (Fig. 3b), except for a statistically significant increase between B2 and B3 (*p* < 0.05).

Concerning *ldha* transcript (Fig. 3c), an increase in the expression levels from b1 to B1 (*p* < 0.001) and from b3 to B3 is detected (*p* < 0.05). After 48 h, both B2 and B3 lungs display a decrease in *ldha* levels compared to B1 lungs (*p* < 0.05). *ldhb* (Fig. 3d) is present in all the stages and time points analyzed, and statistically significant differences were observed only between b1 and B1 lungs (*p* < 0.05).

*pdha* expression levels (Fig. 3e) are moderately maintained among the groups studied. On the opposite, *pdhb* transcript levels (Fig. 3f) increase from b1 to B1 (*p* < 0.05) but decrease between B1 and B3 lungs (*p* < 0.01).

For all transcripts, fold variation was expressed as the change that occurs from 0 to 48 h of explant culture (Figs. 2g and  3g). Major variations were observed in stages b1 and b2 for glucose transporters (*glut1* and *glut3*) and monocarboxylate transporters (*mct1* and *mct8*) (Fig. 2g). Still, the b3/B3 stages exhibited only minor variations in the same groups of transcripts (Fig. 2g). *hk1* and *ldha* showed a potential synergism with symmetric behavior regarding the fold variation (Fig. 3g). *ldhb* revealed a fold variation decrease for all stages, from 0 to 48 h. Still, no major expression variations were observed in *pfk1*, *pdha*, and *pdhb* (Fig. 3g).

### ldha and lhdb exhibit region-specific expression patterns in the embryonic lung

To study the spatial distribution of lactate dehydrogenase, the expression pattern of *ldha* and *ldhb* was characterized by in situ hybridization. Afterward, representative lungs from the three branching stages were processed for histological sectioning.

**Fig. 4:**
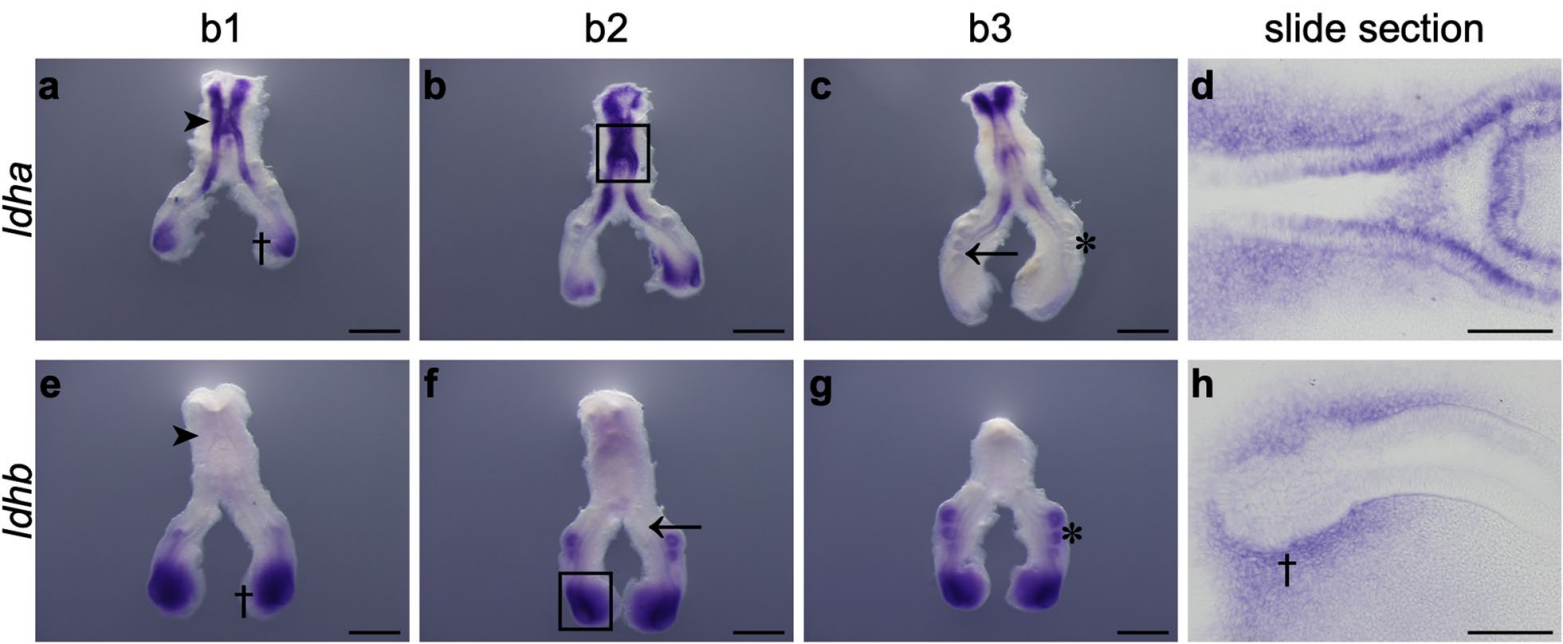
*ldha* and *ldhb* mRNA expression pattern at early stages of chick lung branching. Representative examples of in situ hybridization of stage b1, b2, and b3 lungs for **a-d** *ldha* and **e–h** *ldhb*, n ≥ 9 per stage. Scale bar: whole mount, 500 μm; slide sections, 100 μm. The black rectangle in images **b** and **f** indicates the region shown in the corresponding slide section. Asterisk: secondary bronchi. Black arrow: main bronchus epithelium. Dagger: distal region. Dark arrowhead: trachea region

*ldha* mRNA is present in the proximal epithelium of the lung, namely in the trachea region (Fig. 4a, dark arrowhead; 4b, black rectangle). *ldha* transcript is also expressed in the more distal region of the main bronchus (Fig. 4a, dagger). However, it is not expressed in the main bronchus epithelium and secondary bronchi (Fig. 4c, black arrow and asterisk, respectively). This expression pattern is conserved among the three branching stages but decreases throughout branching morphogenesis, namely for stage b3. Slide sectioning of the hybridized lungs confirmed the presence of *ldha* in both mesenchymal and epithelial compartments of the embryonic trachea (Fig. 4d).

*ldhb* transcript is completely absent from the proximal region of the lung (Fig. 4e, dark arrowhead) and the epithelium of the primary bronchus (Fig. 4f, black arrow). *ldhb* is strongly expressed in the distal-most region of the lung (Fig. 4e, dagger) and the secondary bronchi (Fig. 4g, asterisk). This expression pattern is maintained in the three stages studied. Lung sectioning confirmed that *ldhb* is not expressed in the distal epithelial compartment but exclusively present in the distal mesenchyme of the growing tips (Fig. 4h, dagger).

### Lung active branching sites are associated with high proliferation

**Fig. 5:**
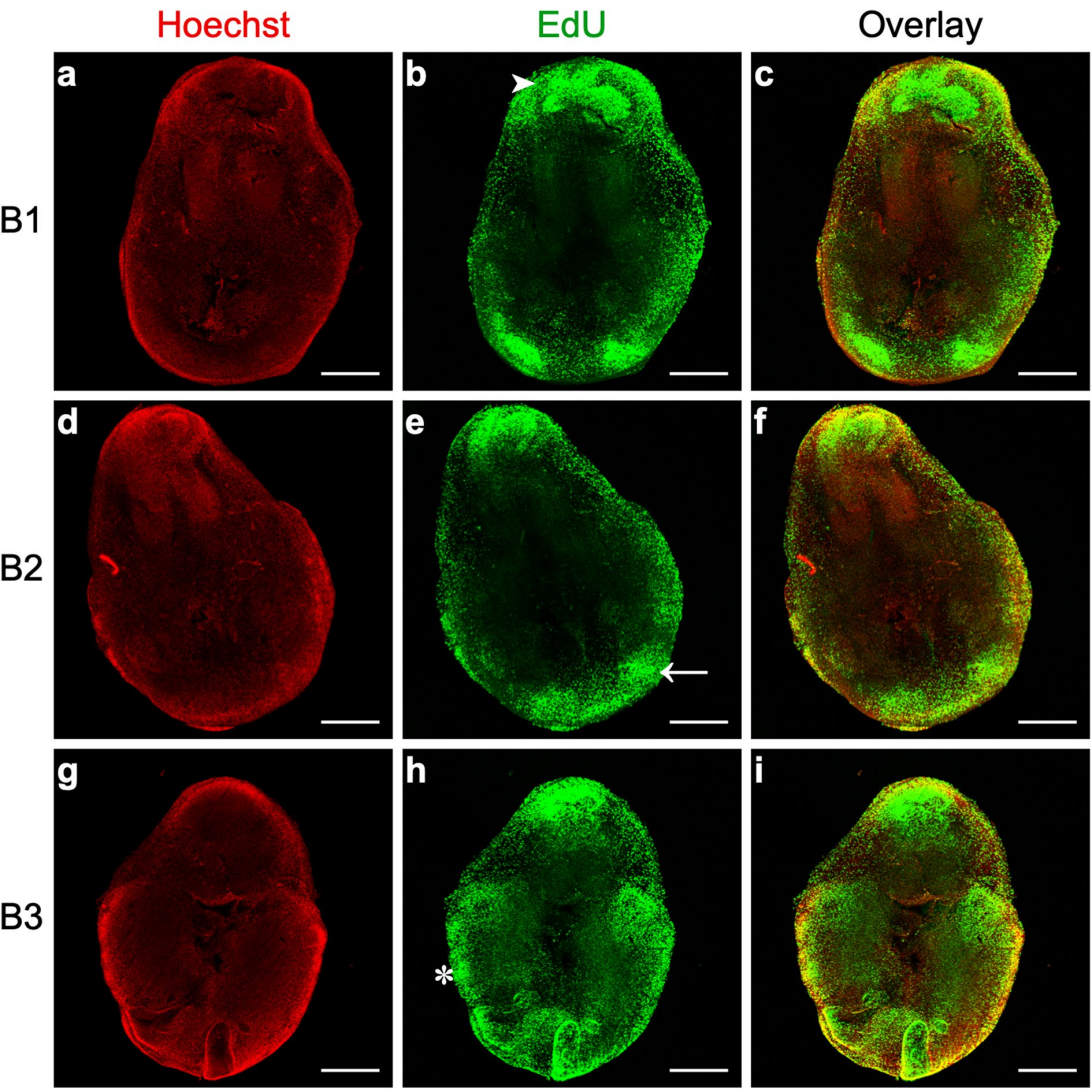
Proliferation analysis of lung branching morphogenesis. Representative confocal microscopy fluorescence images of lungs explants after 48 h in culture (B1, B2, B3). **a**, **d**, **g** Nuclei were stained with Hoechst 33342 (Red). **b**, **e**, **h** Proliferation was assessed using an EdU-based assay; EdU incorporation in DNA was detected using Alexa Fluor 488 (Green). **c**, **f**, **i** Overlay represents the merged images of Hoechst (Red) and EdU (Green). All images are represented as maximum intensity projection of z-stacks. n ≥ 4 per stage. Scale bar: 500 μm. Asterisk: secondary bronchi/active branching sites. White arrow: distal tip. White arrowhead: trachea region

To assess the proliferation status of lung branching morphogenesis, we performed an EdU-based proliferation assay using B1 to B3 lung explants (Fig. 1a). Proliferation was determined by directly assessing EdU incorporation into new DNA strands using Alexa Fluor 488 (Green); nuclei were counterstained with Hoechst 33342 (Red) (Fig. 5).

After 48 h of culture, high proliferation levels were detected in the trachea region (white arrowhead) (Fig. 5b), in the distal tip of the lung (white arrow) (Fig. 5e), and in the secondary bronchi/active branching sites (asterisk) (Fig. 5h). The proliferation patterns are maintained in the three stages (B1, B2, and B3).

### LDH protein levels support the increase of lactate production throughout branching

To understand how LDH protein levels may contribute to the observed increase in lactate production, the protein expression levels of LDHA and total LDH (that recognizes both LDHA and LDHB proteins) were assessed.

**Fig. 6:**
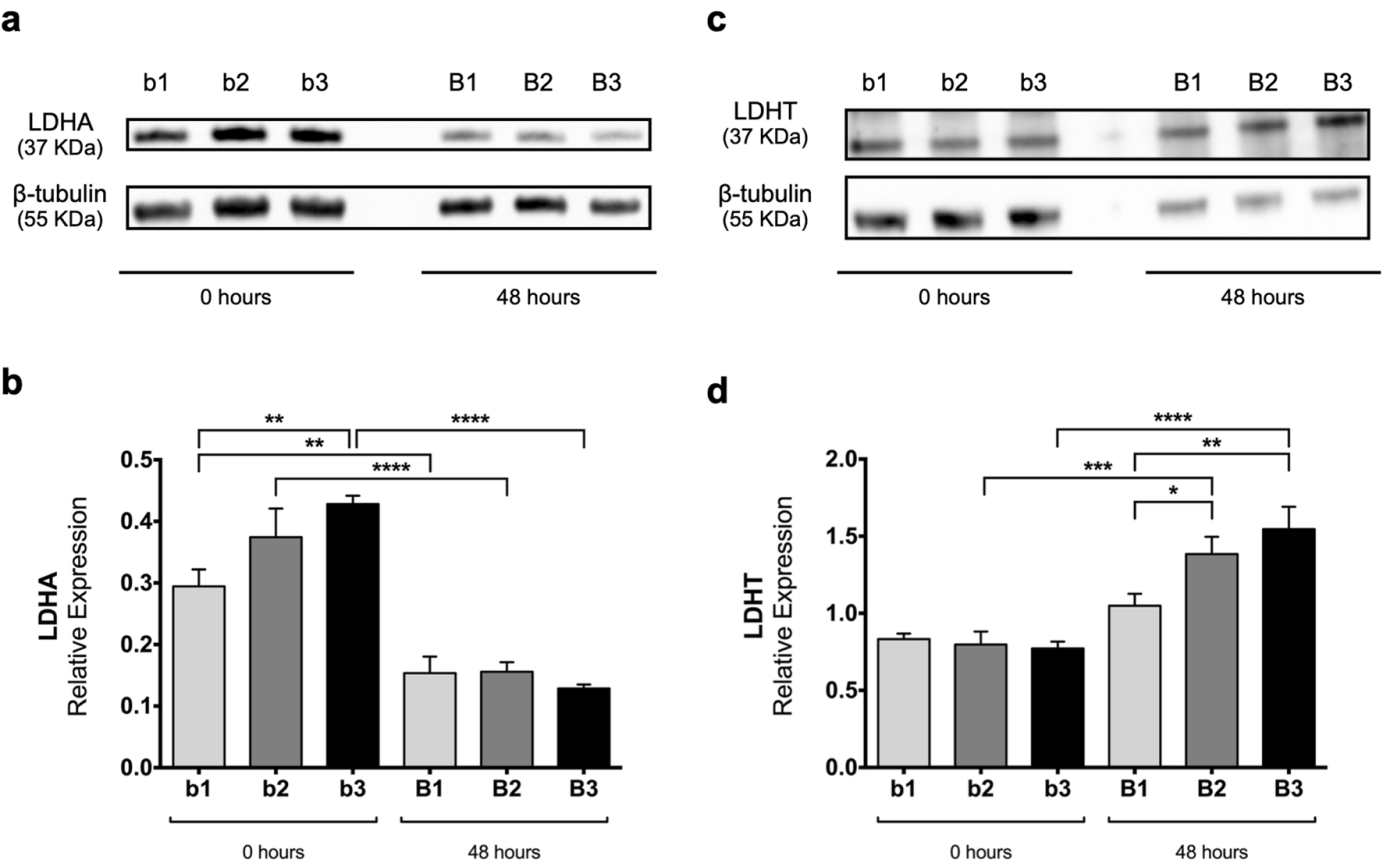
Western blot analysis of LDHA and total LDH throughout chick lung branching. Representative immunoblots for **a** LDHA and **c** LDHT (LDHA and LDHB contribution) of pooled-tissue samples of embryonic lungs at D0 (0 h) (b1, b2, b3) and D2 (48 h) (B1, B2, B3) of explant culture. Loading control was performed using β-tubulin (55 KDa). LDHA and LDHT correspond to 37 KDa. Semiquantitative analysis, of three independent experiments, for **b** LDHA and for **d** LDHT protein expression levels. Results are represented in arbitrary units, normalized for β-tubulin. Results are expressed as mean ± SEM (n = 3/stage/condition). One-Way ANOVA and Fisher’s LSD test were performed. Significantly different results are indicated as: *p < 0.05; **p < 0.01; ***p < 0.001; ****p < 0.0001. Full-length blots are presented in Additional file 1: Figure S2

As seen in Fig. 6a, at 0 h, LDHA protein levels fluctuate between stage/condition. Semi-quantitative analysis (Fig. 6b) revealed that LDHA expression levels increase at 0 h, namely from b1 to b3 (*p* < 0.01). On the other hand, after 48 h of culture, this variation dissipates, and there are no differences between stages. Conversely, when comparing both time points for each stage individually, a statistically significant decrease in LDHA expression levels is observed (Fig. 6b), namely from b1 to B1 (*p* < 0.01), from b2 to B2 (*p* < 0.0001), and from b3 to B3 (p < 0.0001).

In contrast, total LDH (LDHA plus LDHB) displays similar levels between stages at 0 h (Fig. 6c). Semi-quantitative analysis (Fig. 6d) exposed that there are no statistically significant differences between the three stages analyzed at 0 h. After 48 h of culture, total LDH protein levels vary between stages, namely from B1 to B2 (*p* < 0.05) and from B1 to B3 (*p* < 0.01). When comparing both time points for each stage individually, there is a statistically significant increase in total LDH expression levels from b2 to B2 (*p* < 0.001) and from b3 to B3 (*p* < 0.0001); in contrast, from b1 to B1 the protein levels remained unaltered.

### Basal oxygen consumption rate is maintained during branching morphogenesis

**Fig. 7:**
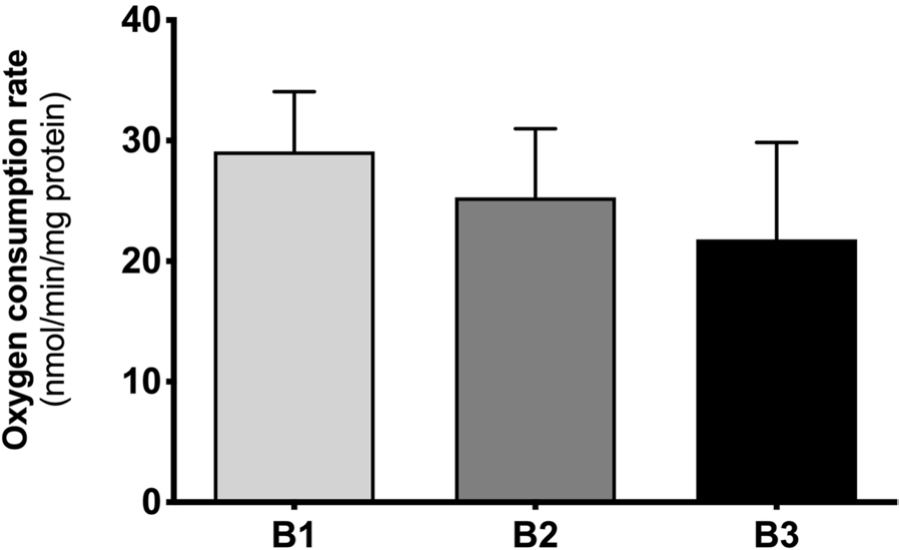
Basal oxygen consumption rate (OCR) measurements throughout early stages of lung branching. Oxygen consumption rate of embryonic lungs after 48 h of explant culture (B1, B2, B3). Results are represented in nmol/min of oxygen consumption normalized for the total amount of protein. Results are expressed as mean ± SEM (n ≥ 5/stage/condition). One-Way ANOVA and Fisher’s LSD test were performed. *p* < 0.05 was considered

To corroborate that the embryonic lung displays a glycolytic preference as branching morphogenesis develops, the respiratory capacity of explant tissue was evaluated. For this purpose, B1 to B3 lung explant tissue was collected after 48 h of culture (Fig. 1a), and the basal oxygen consumption rate was measured (Fig. 7).

After 48 h of culture, basal OCR was maintained between B1, B2, and B3 (Fig. 7). However, a slight decrease tendency in explants’ respiratory capacity throughout pulmonary branching is detected.

## Discussion

Lung development is a complex process characterized by epithelial-mesenchymal interactions mediated by signaling cascades, leading to the formation of a fully functional organ [12, 26]. Altogether, these processes contribute to the formation of a fully functional adult organ responsible for gas exchange.

The metabolic profile of the mammalian adult pulmonary system was studied in the ‘70 and ‘80 s, revealing a metabolism that is primarily dependent on glucose utilization [16–18]. On the other hand, little is known about embryonic lung metabolic needs. The importance of metabolism during development, beyond its canonical role, has been recently reported; several studies have shown that metabolism is highly regulated both in time and space, affecting multiple aspects of animal development [19]. Moreover, glucose uptake and catabolism are essential for all cellular processes and are believed to be enhanced in high proliferative systems such as cancer and embryonic development [20, 27].

Branching morphogenesis is a crucial step in lung organogenesis since it establishes the future airway conducting system [28]. In the chicken lung, b1, b2, and b3 stages correspond to the sequential appearance of the first three secondary buds, with approximately an 8 h gap between them. These morphologically similar stages revealed to be a useful system to study the molecular mechanisms underlying avian branching morphogenesis. Overall, signaling pathways display similar patterns between stages, and the interaction between different signaling events ultimately shapes airway branching [7–9, 11]. In this sense, we wondered if b1, b2, and b3 stages were also similar at the metabolic level or if, despite the molecular similarities, the embryonic lung gradually adapts its metabolic needs to cope with the progressive tissue growth. Accordingly, in this work, we focused on describing the metabolic profile of the embryonic chicken lung during the abovementioned stages of branching morphogenesis. For this purpose, an ex vivo explant culture system was used to precisely measure the metabolic and associated molecular alterations during 48 h of culture [29–31]. This method is particularly relevant to study the whole organ while under very controlled conditions since it preserves the epithelial-mesenchymal interactions that contribute to branching morphogenesis [30, 32]. Lung explants are described to maintain the native physiological interactions between cells and tissues, thus mimicking the in vivo structure and function [29]. The culture medium from ex vivo lung explant culture was collected to study the production/consumption of extracellular metabolites related to glucose catabolism (alanine, lactate, and acetate) by ^1^H-NMR spectroscopy. Afterward, the molecular machinery associated with glucose catabolism (*glut1*, *glut3*, *glut8*, *mct1*, *mct3*, *mct4*, *mct8*, *hk1*, *hk2*, *pfk1*, *ldha*, *ldhb*, *pdha*, and *pdhb*) was characterized by qPCR. *ldha* and *ldhb* mRNA spatial localization was assessed by in situ hybridization. Proliferation status was determined by directly assessing DNA synthesis using an EdU-based assay. LDH protein expression levels were evaluated by western blot. The respiratory capacity of lung explants was evaluated by measuring the basal oxygen consumption rate.

In this study, ^1^H-NMR results revealed that glucose consumption by chicken lung explants decreases from B1 to B2 and from B1 to B3 stage, and remains stable between B2 and B3 stage (Fig. 1c). In B1, glucose consumption is higher, and we believe this is due to a higher nutrient requirement to cope with the very beginning of branching morphogenesis. As lung branching morphogenesis proceeds, glucose consumption profile variation may reflect the need to cope with specific energy and nutrients requirements needed to maintain high proliferative rates. Similarly, the adult mammalian lung metabolic requirements are mainly achieved through glucose catabolism, although other metabolic substrates might support lung energetic demands [16–18, 33].

Glucose cellular uptake relies on the presence of integral membrane glucose transporters (GLUTs) that move glucose from the extracellular space to the cytosol by facilitated diffusion; furthermore, this process depends on the number of glucose transporters expressed in the cell surface. In this sense, the expression levels of selected transporters, *glut1*, *glut3*, and *glut8*, in the chicken developing lung were characterized. *glut1*, *glut3*, and *glut8* are expressed in the three pulmonary stages studied (Fig. 2b–d). After 48 h in culture, major variations of *glut1* and *glut3* are detected in B1 and B2 stages (Fig. 2b, c) but not in the B3 stage (Fig. 2b, c). *glut1* and *glut3* expression level variations may support the observed glucose consumption variations. *glut1* and *glut3* transcripts have already been identified during chicken embryonic development in other tissues than the lung, namely, in the brain, muscle, and heart; moreover, *glut1* and *glut3* expression levels are developmentally regulated in these structures [34]. In the adult, *glut1* and *glut8* are ubiquitously expressed, whereas *glut3* is highly expressed in the brain, similarly to their mammalian counterparts [35–37]. In human fetal lung, GLUT1 is present in bronchial and primitive alveolar epithelial cells during the branching phase but becomes progressively less expressed from week 19 onwards [38]. GLUT1 is widely expressed in adult human tissues, and it is commonly overexpressed in several tumor types [39]. On its turn, GLUT3 is highly expressed in lung cancer and actively contributes to cellular glucose uptake and consequent high proliferation of pulmonary tumor cells [40]. Altogether *glut1*, *glut3*, and *glut8* may likely contribute to sustaining glucose consumption throughout early pulmonary branching morphogenesis stages.

Once inside the cell, glucose is converted into glucose-6-Pi in a reaction catalyzed by hexokinase [41]. *hk1* is expressed in the embryonic chicken lung during branching morphogenesis (Fig. 3a), whereas *hk2* transcript is barely detected (data not shown).

PFK1 catalyzes a key step of glycolysis, the unidirectional conversion of fructose-6-Pi and ATP into fructose-1,6-Pi and ADP [41]. In the embryonic chicken lung, *pfk1* transcript is present in all stages studied without major variations between groups (Fig. 3b). *pfk1* expression levels may contribute to control the relative glucose flux into the glycolytic pathway throughout early branching.

From the glycolytic pathway, each molecule of glucose is converted into two molecules of pyruvate. In our explant culture medium, several pyruvate-derived metabolites (alanine, lactate, and acetate from acetyl-CoA) were detected (Fig. 1d–f). During branching morphogenesis, the embryonic chicken lung progressively increases alanine production that is finally exported to the extracellular medium (Fig. 1d). Previous studies revealed that, in the adult mammalian lung, part of the glucose carbons serves for alanine production through pyruvate transamination [17, 18, 33]. Similarly, in prostate cancer, the production of significantly high levels of alanine supports the need for proliferating cells for protein synthesis and membranogenesis [42]. Moreover, in pancreatic ductal adenocarcinoma, alanine produced from the surrounding environment is used by tumor cells for biosynthetic purposes [43]. Thus, alanine is an important end product of glucose catabolism and, in the developing lung, may contribute to protein biosynthesis to support active pulmonary growth.

Lactate was detected at high concentrations in the explant culture medium. Indeed, in our culture conditions, there is a sharp increase in lactate production from stage B1 to B3, and from stage B2 to B3 (Fig. 1e), concurrently with the increase in branching (Additional file 1: Figure S1). The lactate/glucose ratio revealed that in B1 and B2 explants, 41% and 58% of glucose is directed to lactate production, respectively; in B3 explants, around 76% of glucose is converted into lactate. Likewise, in the mammalian adult lung, around 50% of the glucose carbons are metabolized into lactate [16–18, 33]. Therefore, lactate production by the developing lung may be facilitating the uptake and incorporation of nutrients by promoting the activity of biosynthetic pathways to, for instance, form new biomass and regenerate NAD^+^ required for maximal glycolytic flux [44]. The increase in alanine and lactate production is characteristic of high proliferative systems in which fast energy and macromolecules are necessary to sustain the growth.

Lactate dehydrogenase (LDH) is the enzyme responsible for the interconversion of pyruvate into lactate and NADH into NAD^+^. This enzyme is a tetramer of two types of subunits, LDHA (formerly LDH-M) and LDHB (formerly LDH-H), encoded by *ldha* and *ldhb*, respectively [45]. In this work, we characterized the expression levels of both *ldha* and *ldhb* in the embryonic chicken lung. *ldha* expression increases from b1 to B1 and from b3 to B3 (Fig. 3c). Regarding 48 h’ time point, the expression levels are higher at B1 and then decrease substantially to B2 and B3 (Fig. 3c). On the other hand, *ldhb* expression levels decrease from 0 to 48 h for b1 stage (Fig. 3d). Although the relative expression levels of *ldhb* are greater than *ldha*, higher variations accompanying branching were observed in *ldha* (Fig. 3g). Afterward, we performed in situ hybridization to assess *ldha* and *ldhb* spatial localization. *ldha* and *ldhb* displayed region-specific expression patterns in the chicken developing lung. *ldha* is highly expressed in the proximal region of the lung (Fig. 4a and d) and non-existent in the main bronchus epithelium and secondary bronchi (Fig. 4c). In opposition, *ldhb* transcript is absent from the proximal region of the lung (Fig. 4e) but expressed in the distal region. *ldhb* is specifically expressed in the growing tips and secondary buds (Fig. 4e and g), suggesting an association with highly proliferative regions. Interestingly, in the chicken and mouse embryo, *ldhb* is found mostly in the posterior (distal-most) region of the embryo, the tailbud region [25]. *ldha* and *ldhb* specific patterns remind a spatial distribution typical of proximal–distal markers.

To determine the proliferation status of lung branching morphogenesis, we performed an EdU proliferation assay, using B1 to B3 lung explants (Fig. 1a). High levels of proliferation were detected in the trachea region (white arrowhead) (Fig. 5b), matching *ldha* mRNA expression pattern (Fig. 4d). In addition, high levels of proliferation were also present in the secondary bronchi/active branching sites (asterisk) (Fig. 5h), and in the distal tips of the developing lung (white arrow) (Fig. 5e), which coincides with *ldhb* transcripts spatial localization (Fig. 4e–h). These results point towards an association between *ldha* and *ldhb* expression localization and proliferation events occurring during branching morphogenesis.

After the mRNA expression studies, we performed western blot for LDHA and total LDH (LDHA and LDHB contribution). LDHA displayed a decrease between 0 and 48 h (Fig. 6a, b). In contrast, total LDH expression levels increased from 0 to 48 h of ex vivo culture (Fig. 6c, d). From these results, we speculate that LDHB may be contributing to the increase of total LDH protein levels. When we compare mRNA and protein expression data, an inverse pattern between the same *ldh*/LDH isoform is observed (Figs. 3c vs 6b; Figs. 3d vs 6d). It is generally assumed that mRNA expression levels directly correlate with protein levels. However, several studies have demonstrated that this not always occurs [46, 47]. Distinct factors may account for these differences, such as regulatory mechanisms at transcriptional and/or translational level, or mRNA and protein stability, that can collectively impact total protein levels. In addition, given western blot results and mRNA spatial localization, it is possible that during branching, distal regions produce more lactate to cope with high proliferation rates. The increase in LDH total levels may account for the observed rise in extracellular lactate levels, which seem to be a characteristic of the developing lung.

Since lactate exchange between the cytosol and the extracellular space depends on monocarboxylate transporters (MCTs), we decided to study MCTs in the developing lung. *mct1* and *mct8* expression levels behave similarly throughout branching (Fig. 2e, f) and recapitulate GLUTs mRNA expression behavior. *mct1* displays a considerable increase in stage b1 after 48 h in culture (Fig. 2e), suggesting that it may support intracellular lactate export. Likewise, *mct3* and *mct4* were evaluated but displayed very low expression levels (data not shown). MCT1 has been described in both human and rat fetal lung; additionally, MCT inhibition leads to a decrease in rat lung branching [38]. More recently, Oginuma and colleagues found that *mct1* is expressed, in a graded manner, in the tailbud and posterior PSM of the chicken embryo. Moreover, chicken explants cultured with MCTs inhibitor α-cyano-4-hydroxycinnamic acid (CNCn) displayed increased intracellular lactate levels [48]. On the other hand, cancer cells are equipped with MCTs, mainly MCT1 and MCT4, to exchange lactate and regulate pH homeostasis [49]. MCT4 is the predominant monocarboxylate transporter for lactate export in glycolytic cancer cells; conversely, MCT1 is a passive transporter that can operate in both directions and, in cancer systems, it is described to facilitate lactate export [50]. In fact, MCT1 lactate transport depends on the intracellular/extracellular concentrations of lactate and protons [50]. Chicken *mct8*, also known as thyroid hormone transporter, was previously described in the developing brain, retina, spinal cord, kidney, and testis of the chicken [51, 52]. Moreover, *mct8* knockdown impacts embryonic chicken development, meaning that *mct8* expression is crucial from the very early stages of organogenesis [53]. However, MCT8 does not transport lactate, but it may transport other monocarboxylates and amino acids [54]. In the embryonic chicken lung, *mct1* can be the transporter responsible for cellular lactate exchange.

Pyruvate can be transformed into acetyl-CoA through an irreversible enzymatic reaction catalyzed by pyruvate dehydrogenase (PDH) and then incorporated in the TCA cycle or converted into acetate [41]. Both isoforms of pyruvate dehydrogenase, *pdha* and *pdhb*, are expressed in the embryonic chicken lung (Fig. 3e, f). In the embryonic lung explant culture medium, we did not find detectable levels of metabolites from the Krebs cycle in the ^1^H-NMR spectra. On the other hand, acetate was identified, and its production is increased in later stages of early pulmonary branching (Fig. 1f). Altogether, these data suggest that part of pyruvate may be diverted to acetate, through acetyl-CoA, as pulmonary branching proceeds. In mammals, glucose-derived pyruvate can generate acetate, and this phenomenon is more pronounced when under conditions of hyperactive glucose metabolism [55]. Moreover, in the adult mammalian lung, glucose carbons can incorporate acetate for lipogenesis purposes and serve for surfactant production [17, 18, 33, 56]. Thus, in the embryonic lung, acetate may also point to a hyperactive glucose metabolism and can be produced to incorporate the newly synthesized cellular membranes, a common mechanism of high proliferative cells.

Lastly, to assess whether the embryonic lung truly shows a glycolytic preference during branching morphogenesis, mitochondrial respiration was evaluated. For this purpose, the respiratory capacity of explants was assessed by measuring the basal oxygen consumption rate of the embryonic lungs (Fig. 7). After 48 h of culture, explants display a steady basal OCR in the three stages; nonetheless, there is a slight tendency to decrease as branching morphogenesis proceeds (Fig. 7). This phenomenon occurs in parallel to the progressive increase in alanine, lactate, and acetate production, previously described (Fig. 1d–f). Altogether, these results suggest that, under aerobic conditions and with functional mitochondria, the embryonic lung seems to shift to a glycolytic lactate-based metabolism throughout branching morphogenesis.

## Conclusions

**Fig. 8:**
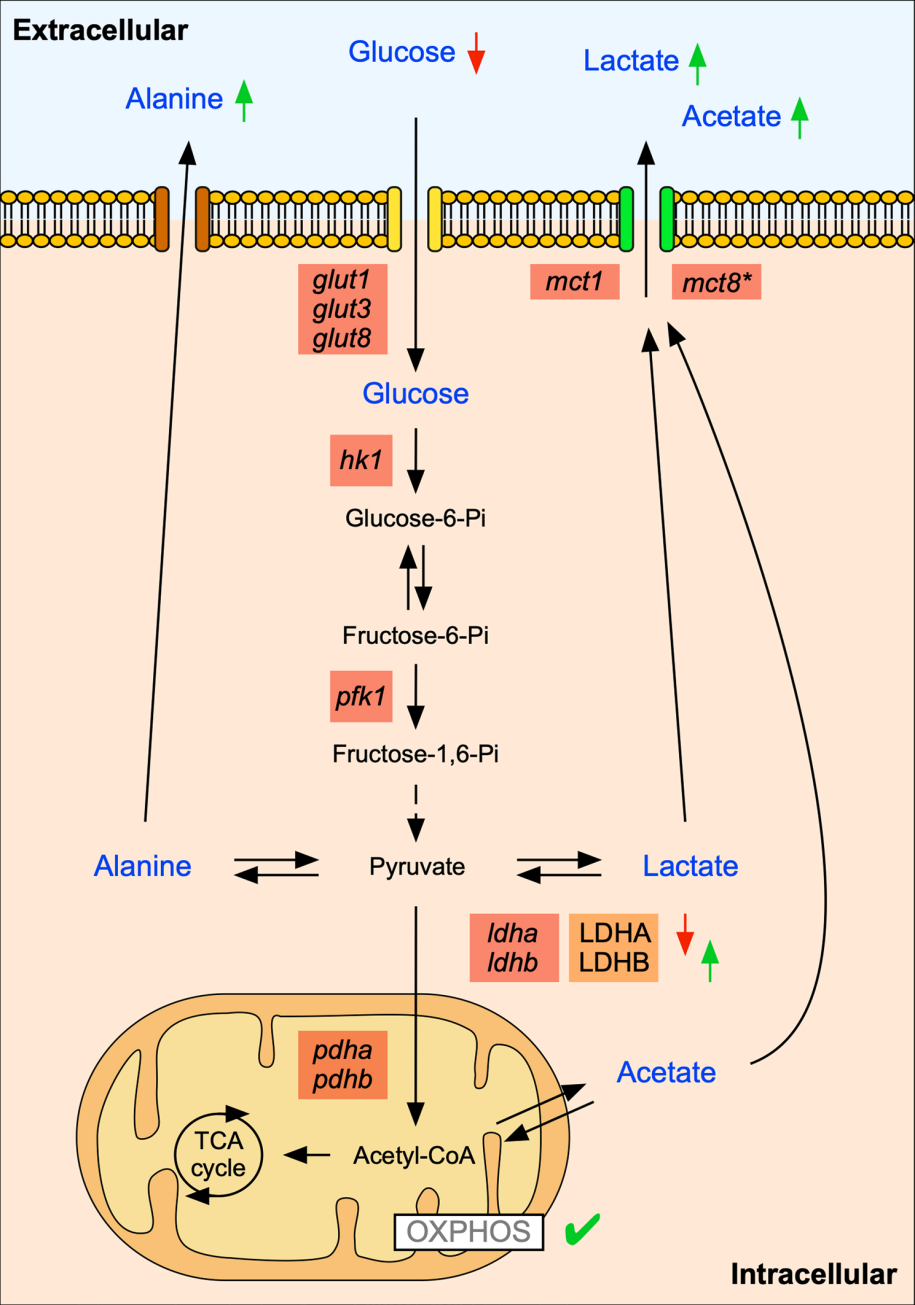
Schematic representation of the metabolic profile of early pulmonary branching morphogenesis. The metabolites detected in the extracellular medium (blue labeling) can either be consumed (glucose) or produced (alanine, lactate, and acetate) by the lung tissue. The symbols refer to increase (↑) or decrease (↓) in the metabolites, considering their particular fate. Temporal metabolite changes are accompanied by variations in the expression of *glut1*, *glut3*, *glut8*, *hk1*, *pfk1*, *mct1*, *mct8*, *ldha*, *ldhb*, *pdha*, and *pdhb*. Decreased LDHA and increased LDHB protein levels may contribute to the observed increase in the extracellular levels of lactate. OXPHOS is active with mitochondria displaying a constant rate of oxygen consumption. The symbols refer to: does not contribute to lactate transport (*); oxidative phosphorylation is active (✓)

This study describes the temporal metabolic changes that occur during early chicken pulmonary branching. Throughout this period, we observed changes in the metabolite profile that occur concurrently with variations in the expression levels of pivotal enzymes and transporters from the glycolytic pathway and with a steady oxygen consumption rate (Fig. 8). It appears that pulmonary branching morphogenesis progressively adapts to a glycolytic lactate-based metabolic profile, suggesting a Warburg-like metabolism. This metabolic rewiring to aerobic glycolysis is also observed in other high proliferative developing systems and allows the production of energy and biomass during embryonic development [20–22, 24, 25, 57]. Moreover, this metabolic adaptation might exert additional signaling functions throughout pulmonary branching, and further studies are required to address this topic. We acknowledge that this study has a strong descriptive perspective, but we are addressing a subject that has not been exploited so far. This report highlights the importance of metabolic regulation during the early stages of lung development and lays the groundwork for future mechanistic studies on this topic.

## Methods

### Ethical statement

This work was performed at the early stages of chicken embryonic development and, therefore, does not require ethical approval following the European Parliament Directive 2010/63/EU of 22 September 2010 and the Portuguese Directive 113/2013 of 7 August 2013 on the protection of animals used for scientific purposes.

### Tissue collection

Fertilized chicken eggs, *Gallus gallus*, were incubated between 4.5 and 5.5 days (Embryonic day 4.5–5.5) in a 49% humidified atmosphere at 37 °C (Termaks KB400, Norway). Embryonic lungs were obtained by microdissection under a stereomicroscope (Olympus SZX16, Japan) and classified in stage b1, b2, or b3 according to the number of secondary buds formed per bronchus; 1, 2, or 3, respectively [7]. Dissected lungs were processed for ex vivo lung explant culture or for in situ hybridization.

### Ex vivo lung explant culture

Chicken lung explant culture was performed, as previously described [30], to study potential temporal metabolic changes while in controlled settings. Briefly, after dissection in PBS, lungs were placed on top of 8 µm nucleopore polycarbonate membranes (Whatman, USA) and incubated for 48 h in 200 µL of medium 199 (5.5 mM glucose; Sigma, USA) supplemented with 10% (V/V) chick serum (Invitrogen, USA), 5% (V/V) heat-inactivated fetal calf serum (Invitrogen), 1% (V/V) L-glutamine (Invitrogen), 1% (V/V) penicillin 5000 IU/mL plus streptomycin 5000 IU/mL (Invitrogen) and 0.25 mg/mL of ascorbic acid (Sigma). The medium was replaced by fresh supplemented medium at 24 h of culture. Lung explants were photographed at 0 h (D0), 24 h (D1), and 48 h (D2) with a camera (Olympus U-LH100HG) coupled to a stereomicroscope (Olympus SZX16). The medium was collected at D0, D1, and D2 for ^1^H-NMR spectroscopy analysis. D0 and D2 lung explants were collected for RNA and protein extraction; D2 lung explants were collected for EdU proliferation assay and basal oxygen consumption rate assay.

### ^1^H-NMR spectroscopy

Samples of 200 µL of medium were collected from ex vivo lung explant culture, at D0, D1, and D2, and analyzed by ^1^H-NMR spectroscopy (n ≥ 5/stage/condition) according to [58]. A Bruker Avance 600 MHz spectrometer with a 5 mm QXI probe and z-gradient (Bruker Biospin, Germany) was used, and spectra accessed at 25 °C. Solvent-suppressed ^1^H-NMR spectra were acquired with 6 kHz spectral width, 14-s interpulse, 3-s water presaturation, 45-degree pulse angle, 3.5-s acquisition time, and a minimum of 128 scans. Sodium fumarate (singlet, at 6.50 ppm) was used as an internal reference at 10 mM. The following metabolites were detected and quantified: H1-α glucose (doublet, 5.22 ppm), alanine (doublet, 1.46 ppm), lactate (doublet, 1.33 ppm), and acetate (singlet, 1.9 ppm). To quantify the relative areas of ^1^H-NMR resonances, the curve-fitting routine supplied with the NUTSproTM NMR spectral analysis program (Acorn NMR, USA) was used. D0 media samples were used as reference/control. The metabolite consumption/production during 48 h of explant culture was calculated following the mathematical formula | (D1-D0) + (D2-D0) |. Metabolite data were normalized to the total amount of protein.

### Quantitative PCR

Total RNA was extracted from D0 and D2 lung explants using TripleXtractor directRNA kit (Grisp, Portugal). RNA integrity and purity were determined using the Experion RNA StdSens Analysis Kit (Bio-Rad, USA), and RNA concentration determined by Nanodrop 1000 Spectrophotometer (Thermo Fisher Scientific, USA). Total RNA was treated with DNase I (Thermo Fisher Scientific) and then reversely transcribed to obtain cDNA using GRS cDNA Synthesis kit (Grisp). Specific exon-exon spanning primers were designed for the amplification of target and housekeeping transcripts (Additional file 1: Table S1). Primers were optimized for annealing temperature and PCR cycles, using NZY Taq 2 × Green Master Mix (NZYTech, Portugal) and, subsequently, for efficiency range. qPCR was performed in duplicate (n ≥ 5/stage/condition) using 1 µL of cDNA and SYBR method according to manufacturer’s instructions, NZY qPCR Green Master Mix (2x) (NZYTech). mRNA expression levels were normalized for both *18s* and *actin-β* housekeeping genes. Data on the gene expression levels were calculated following the mathematical model 2^(-ΔCt) [59].

### Western blot

Pooled samples of D0 and D2 explants (3 pools per stage: 8 lungs per pool) were processed for western blot analysis, as described in [8]. Protein was obtained according to [60]. 10 µg of protein was loaded onto 10% acrylamide minigels, electrophoresed at 100 V in a Mini-PROTEAN Tetra Cell (Bio-Rad), and transferred to 0.2 µm nitrocellulose membranes using a Trans-Blot Turbo Transfer System (Bio-Rad). Blots were probed with primary antibodies for LDHA (1:20,000; #3582, Cell Signaling, USA), for total LDH that recognizes both LDHA and LDHB (1:20,000; #ab52488, Abcam, UK), and for β-tubulin (1:200,000; #ab6046, Abcam) as a loading control. Afterward, blots were incubated with anti-rabbit secondary horseradish peroxidase-conjugated antibody (1:2000; #7074, Cell Signaling). Membranes were developed with Clarity Max Western ECL substrate (Bio-Rad), and the chemiluminescent signal captured using Chemidoc XRS (Bio-Rad). Quantitative analysis was performed with Image Lab software (Bio-Rad). At least three independent experiments per pool were performed (n = 3).

### RNA probes

Total RNA and cDNA from b2 lungs were obtained as previously described. Specific primers were designed for *ldha* (Fw: 5’-GCACTTTCCAAGTAGGTCAAATCC-3’; Rv: 5’- AGTCTTTGGTTTCACGTTGTGT-3’) and *ldhb* (Fw: 5’-GCAGGTTGTTGAAAGTGCCT-3’; Rv: 5’-AGTGAGTAGAGAGCCCACAT-3’). cDNA was used as a template for conventional PCR (GRS Xpert Taq 2 × MasterMix, Grisp). PCR fragments were cloned in the pCR™II-TOPO® vector (TOPO TA Cloning Kit, Invitrogen) and sequenced to determine the insert orientation (GATC Biotech, Germany). *l-cam* RNA probe was produced as previously described [61]. Antisense digoxigenin-labeled RNA probes were produced from PCR-amplified fragments using SP6 or T7 RNA polymerase and according to the manufacturer’s instructions (Roche, Germany).

### Whole mount in situ hybridization

Dissected lungs were fixed in PBS solution containing 4% formaldehyde, 2 mM EGTA, pH 7.5, at 4 °C overnight. Then, the lungs were dehydrated in a methanol series and stored at −20 °C. Tissues were rehydrated through a methanol/PBT series and processed for whole mount in situ hybridization (n ≥ 9 per gene/stage) as previously described by [30]. Briefly, tissues were permeabilized with proteinase K solution (PBT with 0.05% proteinase K) (Roche). PBT washes were made to remove the excess of proteinase K and, tissues were incubated with a post-fixing solution (PBT with 10% formaldehyde, 0.4% glutaraldehyde). After, tissues were incubated with hybridization solution (50% formamide, 6.5% SSC, 1% EDTA 0.5 M pH 9.8, 0.25% t-RNA, 0.2% Tween 20, 0.2% heparin, 0.5% CHAPS), at 70 °C. Then, tissues were incubated with specific probes, in hybridization solution, at 70 °C, overnight. On the following day, several washes were performed with preheated hybridization solution, hybridization solution with MABT (50:50) (5.8% C_4_H_4_O_4_, 4.4% NaCl, 7% NaOH, 1% Tween 20, pH 7.5) and subsequently with only MABT. Next, tissues were treated with blocking solutions [MABT with 20% blocking reagent (Roche); MABT with 20% blocking reagent plus 20% goat serum (Invitrogen)]. Then, lungs were incubated in MABT, 20% blocking reagent, 20% goat serum, and 1:2000 anti-digoxigenin antibody (Roche) solution, overnight. On day 3, the tissues were washed with MABT solution. On the last day, tissues were washed in NTMT solution (0.1 M NaCl, 0.1 M Tris–HCl, 50 mM MgCl_2_, 1% Tween 20) and then incubated in a developing solution (NTMT with BCIP, NBT) (Roche), at 37 °C and protected from light. Each group of lungs/probes was processed simultaneously and developed for the same amount of time. All the lungs were photographed using a camera (Olympus U-LH100HG) coupled to a stereomicroscope (Olympus SZX16).

### Histological sections

Hybridized chicken lungs were dehydrated through increasing ethanol series and embedded in a 2-hydroxyethyl methacrylate solution (Heraeus Kulzer, Germany). 25 µm thick histological slides were produced by a rotatory microtome (Leica RM 2155, Germany). Lung histological sections were photographed using a camera (Olympus DP70) coupled to a microscope (Olympus BX61).

### Proliferation assay and confocal microscopy

After 48 h of culture, half of the explant’s media was replaced by fresh media containing EdU, at a final concentration of 150 µM. Explants were incubated with EdU for 90 min in the same culture conditions. After incubation, tissues were fixed in PBS solution containing 3.7% formaldehyde. Then, tissues were washed in PBS with 3% BSA, and permeabilized in PBS with 0.5% Triton X-100, for 90 min. Tissues were washed again and processed for Click-iT Plus EdU reaction, according to the manufacturer’s instructions (Click-iT™ Plus EdU Cell Proliferation Kit for Imaging, Invitrogen). Detection of the incorporated EdU was performed using Alexa Fluor 488. Nuclei were counterstained with Hoechst 33342 (1:2000). Images were acquired using an Olympus LPS Confocal FV3000 microscope (Olympus).

### Basal oxygen consumption rate measurements

Pooled samples of D2 (2 lungs per pool/per stage) explants were processed to assess respiratory capacity (n ≥ 5/stage/condition). Lung tissue was washed in PBS and incubated, for 2 min, in respiration medium, at 37 °C. Respiration medium was composed by medium 199 (5.5 mM glucose) supplemented with 1% L-glutamine, 1% ITS (0.01 mg/mL recombinant human insulin, 0.0055 mg/mL human transferrin substantially iron-free, and 0.005 μg/mL sodium selenite) (BD Biosciences, USA) and 10 mM HEPES; pH was adjusted to 7.2–7.4, at 37 °C. To assess Oxygen Consumption Rate (OCR), a Clark-type electrode Oxytherm System (Hansatech, UK) was used [62]. Samples were transferred in 1 mL respiration medium to the pre-calibrated and thermostatized (with a water jacket, at 37ºC) electrode chamber. Oxygen recording protocol was performed for 15 min with an open-chamber plus 15 min with a closed-chamber; data acquisition refers to the last 5 min of closed-chamber mode. After each assay, tissue samples were retrieved, washed in PBS, and processed for protein extraction and quantification. Data was analyzed using Oxytrace Plus acquisition software (Hansatech) and normalized to the total amount of protein.

### Statistical analysis

Statistical analysis was performed using GraphPad Prism 6 (GraphPad Software, USA). The normality of distribution was tested using the Kolmogorov–Smirnov test. One-Way ANOVA was performed and followed by Fisher’s Least Significant Difference (LSD) post hoc test for multiple comparisons. All experimental data are presented as mean ± standard error of the mean (SEM) with a statistically significant level of 5% (*p* < 0.05) considered.

## Supplementary Information

**Additional file 1: Table S1.** Primers and qPCR conditions. Primer sequences forward (Fw) and reverse (Rv), corresponding PCR product size, annealing temperature and number of cycles. **Figure S1.** Morphometric analysis of lung explants. **Figure S2.** Uncropped images of LDHA and total LDH immunoblot represented in Fig. 6.

## Data Availability

Not applicable.

## Abbreviations

^1^H-NMR: ^1^H Nuclear Magnetic Resonance
BCIP: 5-Bromo-4-Chloro-3-Indolyl Phosphate
BSA: Bovine Serum Albumin
cDNA: Complementary DNA
D0: 0 Hours
D1: 24 Hours
D2: 48 Hours
EDTA: Ethylenediamine Tetraacetic Acid
EGTA: Ethylene Glycol Tetraacetic Acid
FGF: Fibroblast Growth Factor
GLUT: Glucose Transporter
GLUT1: Glucose transporter 1
GLUT3: Glucose transporter 3
GLUT8: Glucose transporter 8
HEPES: N-2-Hydroxyethylpiperazine-N'-2-Ethanesulfonic Acid
HK1: Hexokinase 1
HK2: Hexokinase 2
L-CAM: Chicken E-cadherin
LDH-H: Lactate Dehydrogenase Isoform from Heart
LDH-M: Lactate Dehydrogenase Isoform from Muscle
LDH: Lactate Dehydrogenase
LDHA: Lactate Dehydrogenase Isoform A
LDHB: Lactate Dehydrogenase Isoform B
LDHT: Lactate Dehydrogenase Total
LSD: Least Significant Difference
MABT: Maleic Acid Buffer with Tween 20
MCT: Monocarboxylate Transporter
MCT1: Monocarboxylate Transporter 1
MCT3: Monocarboxylate Transporter 3
MCT4: Monocarboxylate Transporter 4
MCT8: Monocarboxylate Transporter 8
mRNA: Messenger RNA
NAD^+^: Nicotinamide Adenine Dinucleotide
NADH: Nicotinamide Adenine Dinucleotide
NBT: 4-Nitro Blue Tetrazolium Chloride
OCR: Oxygen Consumption Rate
OXPHOS: Oxidative Phosphorylation
PBS: Phosphate Buffered Saline
PBT: Phosphate Buffered Saline with Tween 20
PCR: Polymerase Chain Reaction
PDH: Pyruvate Dehydrogenase
PDHA: Pyruvate Dehydrogenase Isoform A
PDHB: Pyruvate Dehydrogenase Isoform B
PFK 1: Phosphofructokinase 1
PSM: Presomitic Mesoderm
qPCR: Quantitative Polymerase Chain Reaction
SEM: Standard Error of the Mean
SHH: Sonic Hedgehog
TCA: Tricarboxylic Acid Cycle
WNT: Wingless-related Integration Site
