# Supplementary material for: Lung branching morphogenesis is accompanied by temporal metabolic changes towards a glycolytic preference
Source: Cell Biosci. 2021 Jul 17;11:134. doi: 10.1186/s13578-021-00654-w (PMC8285861; doi:10.1186/s13578-021-00654-w)
Supplement: Supplementary file 1 — Additional file 1: Table S1. Primers and qPCR conditions. Primer sequences forward (Fw) and reverse (Rv), corresponding PCR product size, annealing temperature and number of cycles. Figure S1. Morphometric analysis of lung explants. Figure S2. Uncropped images of LDHA and total LDH immunoblot represented in Fig. 6. [file 13578_2021_654_MOESM1_ESM.pdf]

Supplementary information

Table S1. Primers and qPCR conditions. Primer sequences forward (Fw) and reverse (Rv), corresponding PCR product size, annealing temperature and number of cycles.

Gene	Sequence 5'-3'	Size (bp)	Annealing T (°C)	Cycles
actin-β	Fw – CTTCTAAACCGGACTGTTACCA<br>Rv – AAACAAATAAAGCCATGCCAATCT	100	58	30
18s	Fw – TCTTTCTCGATTCCGTGGGT<br>Rv – AACGCCACTTGTCCCTCTAC	157	58	30
glut1	Fw – GCAGTTCGGCTACAACACCG<br>Rv – ATCAGCATGGAGTTACGCCG	222	58	40
glut3	Fw – GTACCGTTCGGGTTCCGTTAG<br>Rv – AATGGCAGCAACAGAAACAGC	115	62	35
glut8	Fw – AGCTTTGGCTTCGTGCTAGG<br>Rv – GTAGCCTCCCAGTATTCCTCC	153	58	40
mct1	Fw – TCGGAGCCTTCATCTCCATTG<br>Rv – CAATCAAACCACACCCCGAG	234	58	40
mct3	Fw – CATCGGGCTGGTCCTACTTA<br>Rv – GTCTTCTTCCCTCGGTTCAG	183	58	40
mct4	Fw – GGATCTGCACTCAGGGAACC<br>Rv – GGAAAGGCGTAGGAGAACCC	151	62	40
mct8	Fw – TTCTTCTGCTCTCCCATCGT<br>Rv – CGACGCTTGAAGTAGTGACC	218	58	40
hk1	Fw – CTGGCCTACTACTTCACCGAG<br>Rv – TCACTGTCGCTGTTGGGTTA	166	58	35
hk2	Fw – GCGCAGAAGGTGGACAAATAC<br>Rv – TGCCAAGAAGTCTCCGTCCT	192	58	40
pfk1	Fw - CGTGGGAGGAGCTTTGAGAA<br>Rv - CAGCCCACCTCACGTATCTG	236	56	40
ldha	Fw – AAGACGCCGGCAGTACAC<br>Rv – GAGTGTGCAGTCACGCTGTA	285	58	35
ldhb	Fw – ACTTGGTATCCACCCAACCAG<br>Rv – CTCAGCAACGCTAAGACCAAT	244	54	40
pdha	Fw – TCACGGCTTTACCTATGCCC<br>Rv – ACCTGAGCACCGACAATACC	153	58	35
pdhb	Fw – GCTCAGAAGATGCTAAAGGGC<br>Rv – GCTTCTAAACAGTGCCCAACAG	220	58	40

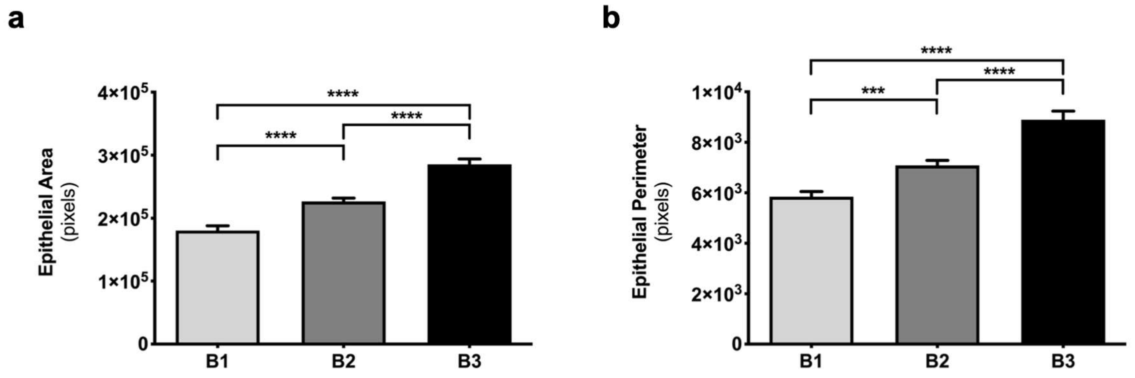

Figure S1. Morphometric analysis of lung explants. The imaging program AxioVision Rel. 4.9.1 (Carl Zeiss, Germany) was used to outline lung explants at 48 hours (B1, B2, B3) and to calculate **a** Epithelial area and **b** Epithelial perimeter. After 48 hours in culture, explants display a progressive increase of the epithelial compartment when compared between stages (B1 *vs* B2 *vs* B3). Results are expressed as mean ± SEM (n>10/stage/condition). One-Way ANOVA and Fisher's LSD test were performed. Significantly different results are indicated as: *** $p < 0.001$ **** $p<0.0001$.

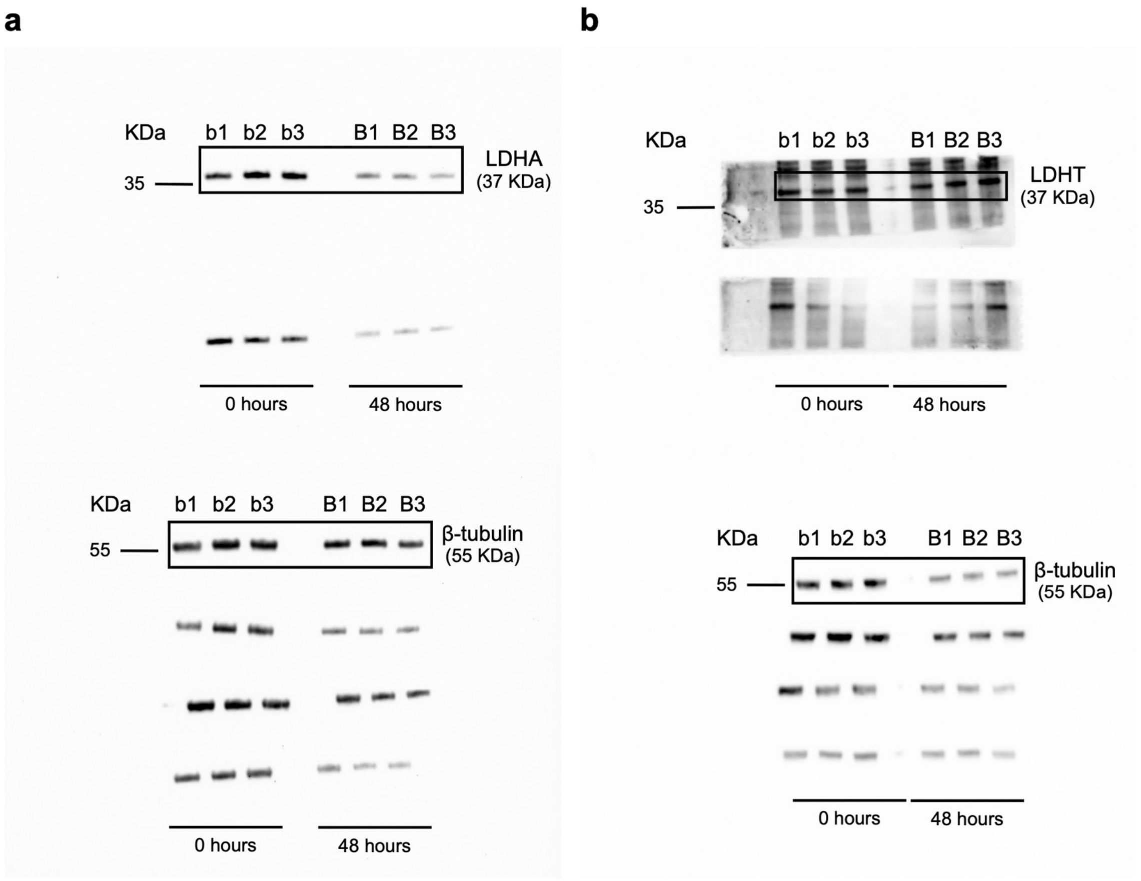

Figure S2. Uncropped images of LDHA and total LDH immunoblot represented in Fig. 6 of the manuscript. Representative immunoblot for **a** LDHA and **b** LDHT (LDHA and LDHB contribution) of pooled-tissue samples of embryonic lungs at 0 hours (b1, b2, b3) and 48 hours (B1, B2, B3) of explant culture. Loading control was performed using β-tubulin, lower panel of the image (55 KDa). LDHA and LDHT correspond to 37 KDa.
